# CMR of LV non-compaction cardiomyopathy: association of clinical presentation and prognosis with cardiac phenotype

**DOI:** 10.1186/1532-429X-13-S1-P291

**Published:** 2011-02-02

**Authors:** Mushabbar A Syed, Steve W Leung, Samy Claude Elayi, Richard J Charnigo

**Affiliations:** 1 Loyola University, Stritch School of Medicine, Maywood, IL, USA; 2 National Institutes of Health, Bethesda, MD, USA; 3 University of Kentucky, Lexington, KY, USA

## Background

Left ventricular non-compaction (LVNC) is a rare congenital disorder characterized by two layered myocardium; trabeculated (non-compacted) and a non-trabeculated (compacted). LVNC is increasingly being recognized due to better imaging technology as a cause for heart failure and sudden cardiac death; however, data on clinical and imaging characteristics remains limited.

## Objective

To investigate the association of clinical presentation and outcomes in LVNC with cardiac phenotype by CMR.

## Methods

Fourteen patients (mean age 33.1 ± 17.6 years, 9 male) were retrospectively identified from CMR database between December 2007 and May 2010. CMR imaging included SSFP cine in standard views and late gadolinium enhancement. Quantitative analysis included left and right ventricular function, volumes, mass, LV wall motion score and non-compacted to compacted myocardium (NC/C) ratios in different segments. Number of involved LV segments and regions of maximum NC/C ratio were also recorded.

Patient's medical records were reviewed for clinical history including NYHA functional class, ECG, telemetry, Holter/event monitoring and electrophysiology studies.

Non-parametric U test, logistic regression analysis and parametric T-test were used to determine statistical significance as appropriate.

## Results

Seven patients presented with acute heart failure including one in cardiogenic shock. Three patients presented with syncope, one with documented ventricular tachycardia (VT).

Mean LVEF was 36.2 ± 22.8% and mean RVEF 31.5 ± 16.7%. LVEF <50% was present in 8 patients (57.1%), RVEF <40% in 7 (50%) and both in 6 (42.8%) patients. Mean NC/C myocardium ratio was 3.7±0.8 with mean of 5.5±3.1 LV segments involved. Patients with LV dysfunction were older, more symptomatic with higher NYHA class, had more myocardial segments involvement with non-compaction, and higher NC/C ratios (Table 1). No myocardial infarction or mid-wall fibrosis was seen on late gadolinium enhancement. One patient had thrombus in the right ventricle associated with severe RV dysfunction.

**Table 1:** 

	LVEF >50% (N=6)	LVEF ≤50% (N=8)	p-value
Age	21.6±11.0	42.5±16.6	0.008
NYHA class	1 (IQR 1-1)	3 (IQR 2-4)	0.007
LV end-diastolic volume index (ml/m^2^)	96.1±15.3	155.4±36.6	0.001
LV end-systolic volume index (ml/m^2^)	38.6±7.5	129.6±38.2	<0.001
LV mass index (g/m^2^)	55.4±8.5	80.2±17.3	0.004
Wall motion score index	1.0±0.02	2.3±0.3	<0.001
Non-compacted segments	3.8±2.1	6.8±3.2	<0.001
NC/C ratio (maximum)	3.2±0.6	4.1±0.8	<0.001
RV EF (%)	44.5±6.8	21.8±15.3	0.002
Heart failure	0 (0%)	7 (88%)	0.005
Any arrhythmia	2 (33%)	5 (63%)	0.592
Ventricular tachycardia	1 (17%)	3 (38%)	0.580

Four patients had non-sustained monomorphic VT. Two patients had premature ventricular complexes on telemetry. One patient had paroxysmal atrial fibrillation, and one had atrioventricular nodal reentry tachycardia (AVNRT).

There were no deaths over a mean follow-up of 7.0 ± 4.6 months. One patient received a heart transplant for severe refractory heart failure. Five patients received an ICD; 4 with non-sustained VT and 1 with severe LV dysfunction. Patient with AVNRT underwent successful ablation.

## Conclusion

Patients with LVNC have a spectrum of cardiac phenotypes ranging from normal LV and RV to severe biventricular dysfunction. Clinical presentation and symptoms are associated with degree of non-compaction and ventricular dysfunction.

